# Robust neuronal differentiation of human iPSC-derived neural progenitor cells cultured on densely-spaced spiky silicon nanowire arrays

**DOI:** 10.1038/s41598-021-97820-4

**Published:** 2021-09-22

**Authors:** Jann Harberts, Malte Siegmund, Matteo Schnelle, Ting Zhang, Yakui Lei, Linwei Yu, Robert Zierold, Robert H. Blick

**Affiliations:** 1 Center for Hybrid Nanostructures, Universität Hamburg, Luruper Chaussee 149, 22761 Hamburg, Germany; 2 School of Electronics Science and Engineering, Nanjing University, Nanjing, 210093 China; 3 Material Science and Engineering, College of Engineering, University of Wisconsin-Madison, Madison, WI 53706 USA

**Keywords:** Biomaterials, Biotechnology, Stem cells, Materials science, Nanoscience and technology

## Abstract

Nanostructured cell culture substrates featuring nanowire (NW) arrays have been applied to a variety of basic cell lines and rodent neurons to investigate cellular behavior or to stimulate cell responses. However, patient-derived human neurons—a prerequisite for studying *e.g.* neurodegenerative diseases efficiently—are rarely employed due to sensitive cell culture protocols and usually long culturing periods. Here, we present human patient induced pluripotent stem cell-derived neurons cultured on densely-spaced spiky silicon NW arrays (600 NWs/ 100 µm$^2$ with NW lengths of 1 µm) which show mature electrophysiological characteristics after only 20 days of culturing. Exemplary neuronal growth and network formation on the NW arrays are demonstrated using scanning electron microscopy and immunofluorescence microscopy. The cells and neurites rest in a fakir-like settling state on the NWs only in contact with the very NW tips shown by cross-sectional imaging of the cell/NW interface using focused ion beam milling and confocal laser scanning microscopy. Furthermore, the NW arrays promote the cell culture by slightly increasing the share of differentiated neurons determined by the quantification of immunofluorescence microscopy images. The electrophysiological functionality of the neurons is confirmed with patch-clamp recordings showing the excellent capability to fire action potentials. We believe that the short culturing time to obtain functional human neurons generated from patient-derived neural progenitor cells and the robustness of this differentiation protocol to produce these neurons on densely-spaced spiky nanowire arrays open up new pathways for stem cell characterization and neurodegenerative disease studies.

## Introduction

Human induced pluripotent stem cells (iPSCs) hold a huge potential for clinical research and application since for example ethical controversies of embryonic stem cells and limited availability of primary human cells required for *e.g.* high-throughput drug screens are overcome^1^. Furthermore, human pathophysiological conditions including neurodegenerative diseases like Alzheimer’s or Parkinson’s can be studied more efficiently by patient-specific iPSCs^2^. The broad availability of human cells for drug development also reduces the high failure rate of clinical translation caused by differences of disease-associated pathways between human and animal cells^3^. At present, all major brain cell types can now be differentiated from iPSCs, and both neuroscience research and clinical translation is facilitated by increasingly complex co-cultures, organoid systems, and blood-brain barrier models^4,5^. The cellular identity of iPSC-derived cell types is usually assessed by quantitative real-time polymerase chain reaction or immunocytochemistry. Additionally, the functional state of the iPSC-derived neurons is characterized by electrophysiological measurements combined with standard compounds which block key receptors, ion channels, and transporters^6^. By default, planar Petri dishes and multiwell plates are used to perform such characterization experiments. However, novel strategies to investigate iPSC-derived cell types could be explored by employing micro- and nanostructured functionalized biocompatible (semi-conductor) materials as cell culture substrates^7,8^.

The influence of the substrate’s chemical and topological properties on biological cells has been investigated for example with respect to adhesion, proliferation, viability, migration, and guiding of seeded cells^9–13^. In this context, so-called nanowire (NW) arrays which feature upright arranged high aspect ratio nanostructures play an increasingly important role^14,15^. By adjusting the length and diameter of the NWs as well as the array pitch, the interaction between cell and NWs can be tuned and different settling regimes have been modeled by Buch-Månson *et al.*^16^. Biological parameters such as cellular growth, viability, morphology, adhesion, and mechanotransduction machinery can be influenced^17–21^, while electrophysiological properties are maintained^22^. Furthermore, NW arrays have been used to measure mechanical cell properties^23^, to interact with the cell’s nucleus^24^, to constrain cell movement and spreading^25,26^, or to direct cell polarization such as the outgrowth of neurites^27,28^. Apart from passive applications, NW arrays have been employed to incorporate an executing interaction such as cell transfection^29^, drug delivery^30,31^ as well as electrical stimulation and sensing^32^. Moreover, integrated *p-n* junctions or the capability of NWs to guide or to emit light might be used for photo-current stimulation^33,34^ as well as for biosensing^35,36^ and potentially in optogenetics^37^.

The majority of the existing studies employ only conventional human cell lines (*e.g.* HEK293, HeLa) or primary neurons from rodents which limits the pertinence of these studies for instance to address human neurodegenerative diseases^38,39^. Indeed, Liu *et al.* were able to demonstrate, that human iPSC-derived cortical neurons can be cultured on small-scale NW arrays with an area of 32×32 µm$^2$^32^, but a drawback of the differentiation protocol was the requirement to culture for at least 65 days to create functional neurons. Aside from that, neuronal differentiation might be altered on large-scale NW arrays, as it has been shown that NW forests can influence intracellular signaling, gene regulation, or basic cell differentiation^40–43^. Moreover, material cues have been discussed as a potent regulator for epigenetics and stem cell function^44^. Thus, changes during terminal differentiation of human iPSCs into neurons might emerge as well on NW arrays and the very same substrate could potentially become useless for most of the promising applications introduced before. Recently, we demonstrated that human iPSC-derived neurons can be generated within 30 days even on large-scale silicon nitride NW arrays with no difference to planar control^45^.

In this work, we present human iPSC-derived neurons cultured on densely-spaced spiky silicon NW arrays with electrophysiologically mature properties in less than 20 days. Additionally, the share of MAP2 positive cells is not only maintained but slightly increased by reducing the number of undifferentiated neural progenitor cells which are used as an intermediate step of the neuronal differentiation. Immunofluorescence (IF) widefield microscopy is used to quantify the cell viability and the number of progenitors and neurons. The cell/NW interface is investigated by scanning electron microscopy (SEM), focused ion beam (FIB) milling as well as confocal laser scanning microscopy (CLSM). A fakir-like interaction is revealed in which the cells only interact with the NW tips. Electrophysiological characteristics are identified with patch-clamp measurements in both current- and voltage-clamp mode and prove the functionality of the neurons. We believe that the short culturing period to generate functional human iPSC-derived neurons and the use of silicon—the standard semiconductor material—for the NW arrays make our platform a promising candidate for future large-scale and high-throughput applications such as retina implants^46^ or neuronal interfaces^47^ and to study pharmacological interventions and cell-intrinsic pathophysiological processes^48^.

## Results and discussion

**Figure 1:**
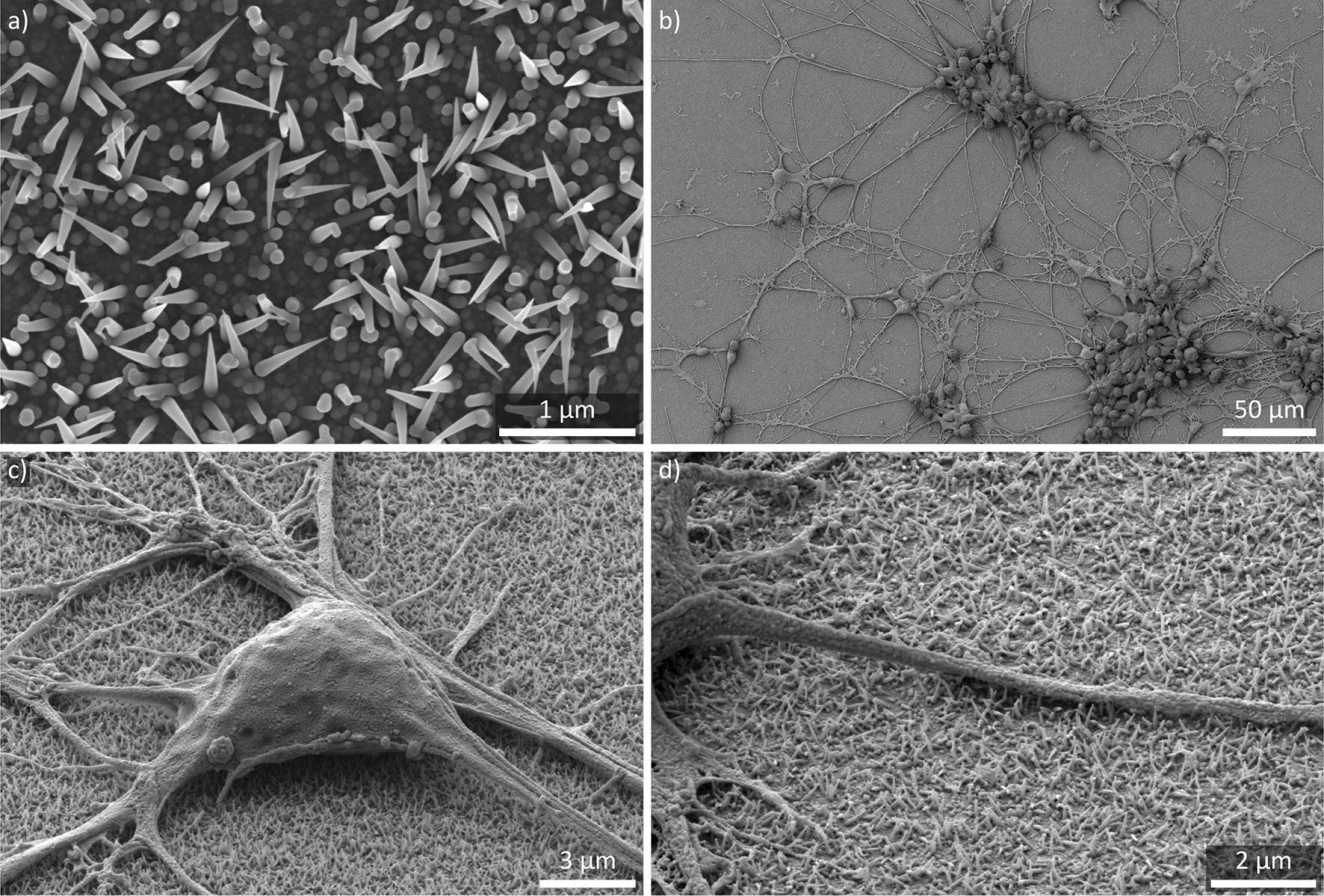
SEM images of a NW sample and human iPSC-derived neurons cultured on NWs. (**a**) Top view of the utilized NW substrate. The NW density is about 600 NWs/100 µm$^2$. The mean NW length is about 1 µm featuring a gradually tapered shape. (**b**) Top view of a neuronal network formed by human iPSC-derived neurons on a NW substrate. (**c**) Cell soma of a human iPSC-derived neuron cultured on NWs. Tilt is 54°. (**d**) Close-up view of a neurite growing on NWs. Tilt is 54°.

A top view SEM image of the silicon NW arrays used to culture the human iPSC-derived neurons is shown in Fig. 1a. The about 1 µm long NWs feature a gradually tapered shape resulting in a spiky tip of about 30 nm. The nanostructures are randomly distributed on the substrate with a density of about 600 NWs/100 µm$^2$ which corresponds to a high NW density compared to other NW substrates used in the field^16^. Random arrangement and random angle orientation are caused by the self-assembling growth process^49,50^. Choosing silicon as the basic element for the substrates not only allows for accessing years of experience in nanofabrication in science and industry but also represents the promising nature of nanoscale silicon for subcellular interfaces^51^.

The protocol to generate the human iPSC-derived neurons used in this study employs proliferating small molecule neural progenitor cells (smNPCs) as an intermediate differentiation step and is based on work from Reinhardt *et al.*^52.^. The smNPCs are maintained in regular well plates coated with Matrigel and are pre-differentiated for 6 days before plating onto the silicon NW arrays or glass coverslips for control (both also coated with Matrigel). First, we investigated the neuronal growth on the NW samples using SEM imaging (Fig. 1b). Overall, the observed neuronal network formation is similar to literature as neurons are growing both separately and in clusters, and neurites build a dense grid in between the neurons^18,22^. The tilted close-up views in Fig. 1c,d display the soma of a neuron and a neurite growing on the NWs. Notably, the cell material is barely in contact with the substrates and only touches the very tip of the NWs. Such a fakir-like settling state on a bed-of-nails was to be expected due to the high NW density^16^. Furthermore, strong interaction with the substrate in form of multiple extensions from the cell soma has been reported before^53^.

**Figure 2:**
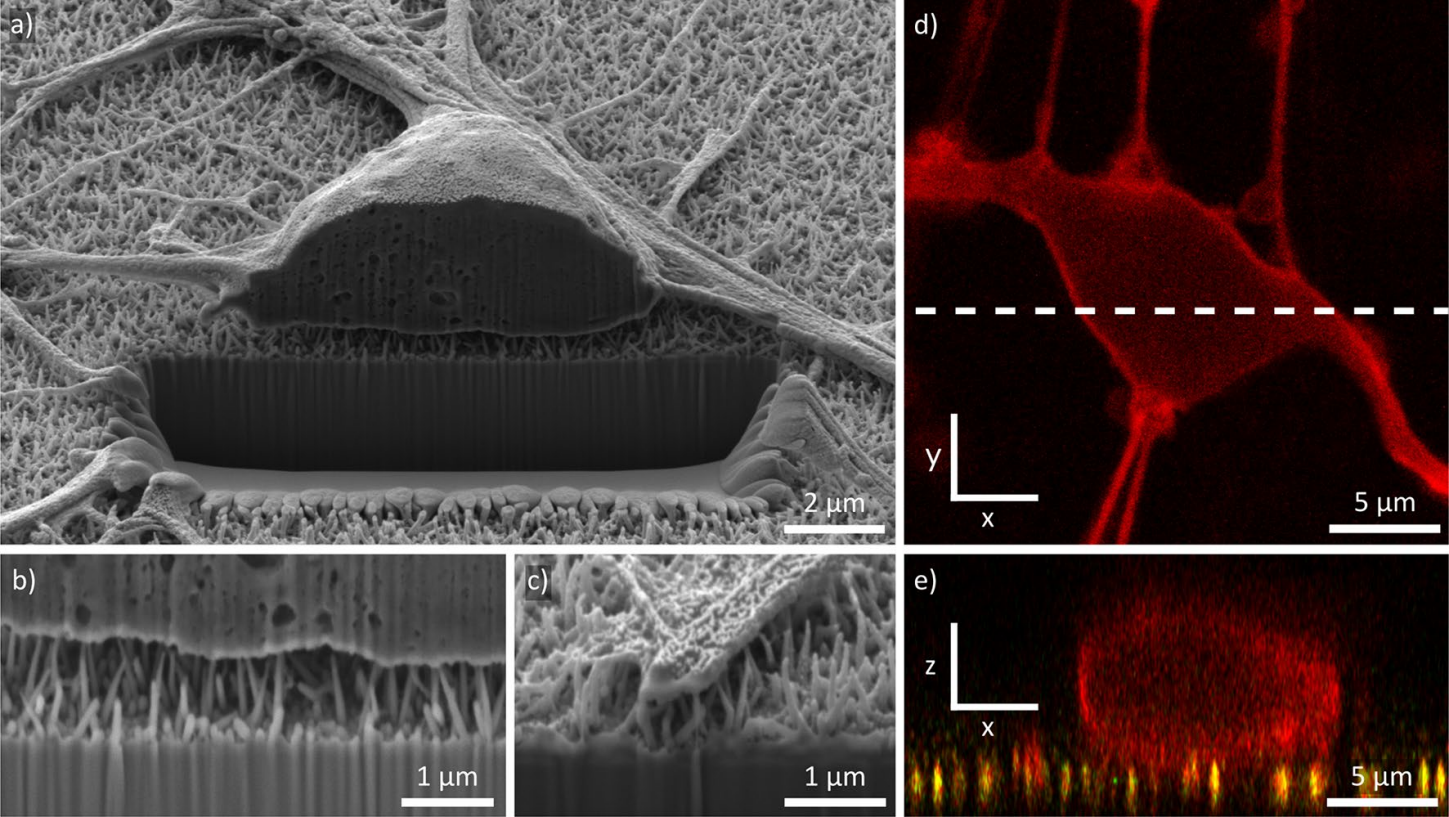
Cross sections prepared by FIB milling and CLSM. (**a**) Cross-sectional SEM image of the cell soma prepared by FIB milling. Tilt is 54°. (**b**, **c**) Close-up SEM images of the NW/cell membrane and NW/neurite interface, respectively. Both cell and neurite are hovering in a fakir like state on the NW tips. The distance to the substrate bottom is approx. 1 µm–the mean length of the NWs. Tilt is 54°. (**d**) Top view of the cell soma imaged via CLSM. The cell membrane is false colored in red. The dashed line indicates the position of the cross section in (**e**). (**e**) Cross section of the cell soma (red) in the x-z plane reconstructed from z-stacks prepared by CLSM. The NW tips’ reflections are false colored in yellow and are in direct contact with the cell membrane.

Cross-sectional images prepared by FIB milling and CLSM were used to support the previous statements regarding the cell’s interaction with the substrate (Fig. 2a–e). Figure 2a displays an overview image of the neuron after FIB milling. The close-up views on the cell soma and a neurite in panel b and c clarify that the cell membrane is hovering approximately 1 µm above the substrate bottom which corresponds to the average NW length. Comparable behavior of cells cultured on high NW densities has been reported before by using FIB-SEM imaging^54^. A similar conclusion can be drawn from z-stacks prepared by CLSM imaging. Figure 2d displays a top view of a neuron cultured on a NW substrate. The cell membrane has been labeled using a membrane stain (false-colored in red). The dashed line indicates the position of the cross section in the z-x plane shown in Fig. 2e at which the cross section was prepared from a stack of images at different z-levels. The reflection of the NWs is false-colored in yellow and originates only from the NWs but not from the Matrigel as demonstrated by imaging an uncoated NW sample (supporting Fig. S1). A corresponding image of a neuron on a control substrate is shown in the supporting material (Fig. S2). The cross-sectional reconstruction confirms the close contact of the cell membrane to the NWs and a fakir-like settling state which is in accordance with literature that studied cells on high-density NW arrays with CLSM^45^. Additionally, we tested for focal adhesions (FAs) to check for interactions with the substrate after long-term culture (11 days after plating). In this regard, we see no influence of the substrate compared to control which demonstrates a generally equivalent culturing environment for our human iPSC-derived neurons (Fig. S3, paxillin positive area (FAs) normalized to the area covered with cells (f-actin/cytoskeleton *via* phalloidin)) and is in agreement with literature for high NW densities^26^. Yet, cell culture substrates with Si nanoneedles have been reported to reduce the formation of FAs in short-term culture (24 h) of mesenchymal stem cells^55^. Nonetheless, the close contact between cell and NW, and the conical shape of silicon nanostructures are able to facilitate cell transfection^29^ and delivery of biological payloads^30^, respectively, as shown in recent work using basic cell lines and mesenchymal stem cells. We believe that comparable applications could also be adapted to our neurons to introduce new pathways in stem cell research.

**Figure 3:**
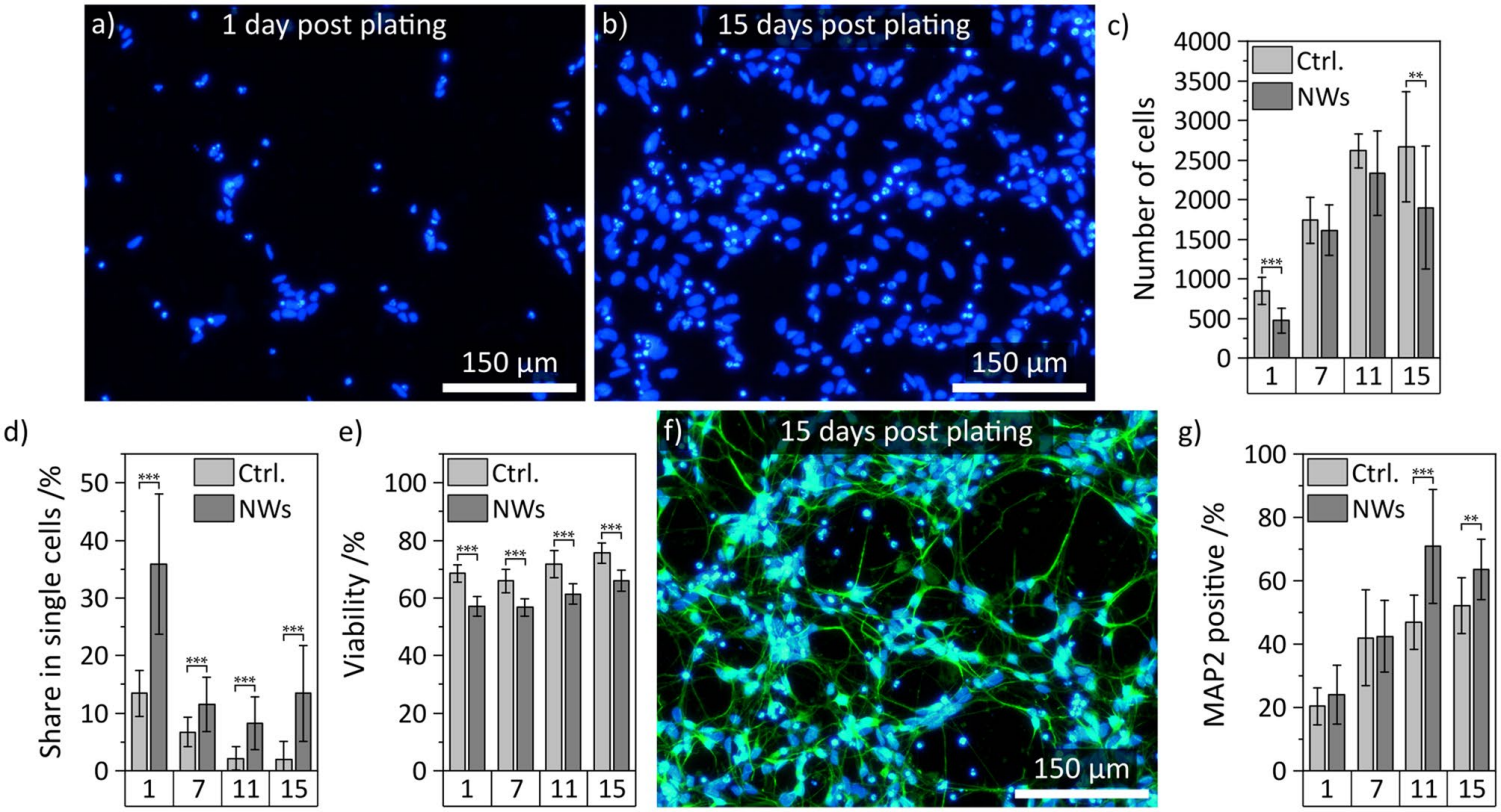
Analysis of cell number, cell viability and neuronal differentiation on NWs compared to planar control (1, 7, 11, and 15 days post plating). (**a**) and (**b**) Exemplary widefield microscopy images of cells 1 and 15 days post plating on NW substrates. The nuclei are labeled in blue using Hoechst 33342. (**c**) Quantification of the cell numbers per image 1, 7, 11, and 15 days after plating compared to control. (**d**) Share of cells not associated with a cell cluster of three or more cells. (**e**) Cell viability 1, 7, 11, and 15 days after plating compared to control. (**f**) Exemplary immunofluorescence image of human iPSC-derived neurons 15 days after plating. The neurons are labeled with MAP2 (green) and counterstained with Hoechst (blue). (**g**) Share in MAP2 positive cells 1, 7, 11, and 15 days after plating compared to control. All error bars are standard deviations (SDs). Statistical significances were calculated using a Mann-Whitney U test: *$P<$ 0.05, **$P<$ 0.01, ***$P<$ 0.001, *cf.* Table S1 of the supporting material.

In the next step, we analyzed the cellular outgrowth using (immuno-) fluorescent labeling and widefield microscopy. Six days pre-differentiated smNPCs were transferred to the NW substrates and snapshots of the neuronal development were taken 1, 7, 11, and 15 days after seeding. Figures 3a,b display exemplary images of Hoechst 33342-stained cell nuclei 1 and 15 days post plating. The total cell number increased significantly over time since terminal neuronal differentiation is a gradual process and part of the cell population is still proliferating. We quantified the increase of the cell numbers and compared the results to planar control (Fig. 3c). The number of cells approximately quadruples within 11 days until it reaches a plateau. The plateauing cell numbers on NWs are slightly smaller in comparison to the control but only the final cell number on NWs–in mean about 2000 and 2500 cells per image, respectively–is significantly different. However, a reduced cell number is overall in agreement with literature reporting on reduced cell proliferation on high-density NW arrays^25^. A visual inspection of the cell spreading on NWs compared to control depicts a considerable increase in cluster formation on control substrates (supplements: Fig. S4). Quantification of the cell clusters using a *Density-Based Spatial Clustering of Applications with Noise* (DBSCAN) algorithm validates the intuitive conclusion and shows that the share of single cells is significantly higher on NW substrates compared to control (*e.g.* about 35% and 13% at 1 DIV, respectively, Fig. 3d). One cause for cluster formation is cell movement and thus, hindered cell movement on the nanostructures is indicated by the increased share in single cells on NWs which is sustained at 7, 11, and 15 DIV. Impaired cell mobility has been described before in literature for cells cultured on NW arrays^19^. The absolute shares of cells in clusters, however, reduce over time on both types of substrates due to continuing proliferation which generates additional cell clusters. The mean cell viability of about 60% on NW arrays is stable over time (*cf.* Fig. 3e) which is in good agreement with literature where high-density NW arrays have no detrimental effect on cell viability^17^. The slight increase in cell viability with further culturing time is reasonable since new vital cells are produced during proliferation. Compared to the control, the cell viability on NWs is lower since fewer new vital cells are produced as described earlier. In addition, we studied the neuronal differentiation of the smNPCs into neurons by IF microscopy. Figure 3f illustrates an exemplary recording of neurons 15 days post plating labeled with an anti-microtubule-associated protein 2 marker (MAP2, green) to identify neuronal phenotype and Hoechst as counterstain (blue). Similar micrographs from a control substrate and 1, 7, and 11 DIV post plating on NWs are available in the supporting material (Fig. S5). As priorly shown via SEM, an intricate neuronal network is developed on the NW arrays. Next, we determined the share of MAP2 positive vital cells 1, 7, 11, and 15 days post plating shown in Fig. 3g. Within this period, the ratio of MAP2 positive cells on NWs increases from about 20% to 60%, which is higher than on control substrates (approx. 50%). The higher percentage in neurons on NW samples could be explained by the slightly reduced proliferation of remaining smNPCs producing new undifferentiated cells. As a result, the smNPCs not only differentiate equally on NWs but also the NWs have a supportive effect to increase the share of terminally differentiated cells. Positive influence on differentiation has also been described in the literature where NW arrays enhanced differentiation *e.g.* for human embryonic stem cells toward definitive endoderm or neuron-like differentiation of mesenchymal stem cells^42,43^. Furthermore, we quantified the neuronal networks between MAP2 positive cells to evaluate the neurite health (supporting Fig. S6). The total network length per image increases from about 10 mm on day 1 to a steady value of 70 mm at days 11 and 15 where neurons cultured on NWs as well as on control show similar network sizes (panel a). Over time, the mean neurite length of individual neurites is slightly reduced on both NWs and control from about 120 µm to 80 µm (panel b) since the MAP2 positive cells become denser and neurites interlink more likely with other neurons. To conclude, even if the cell number and the viability on NW samples are slightly suppressed compared to planar control, the reduced amount of cell clusters and the larger portion of neurons renders the combination of silicon nanowire substrates with neuronal differentiation of smNPCs a promising approach for studying artificial neuronal networks.

**Figure 4:**
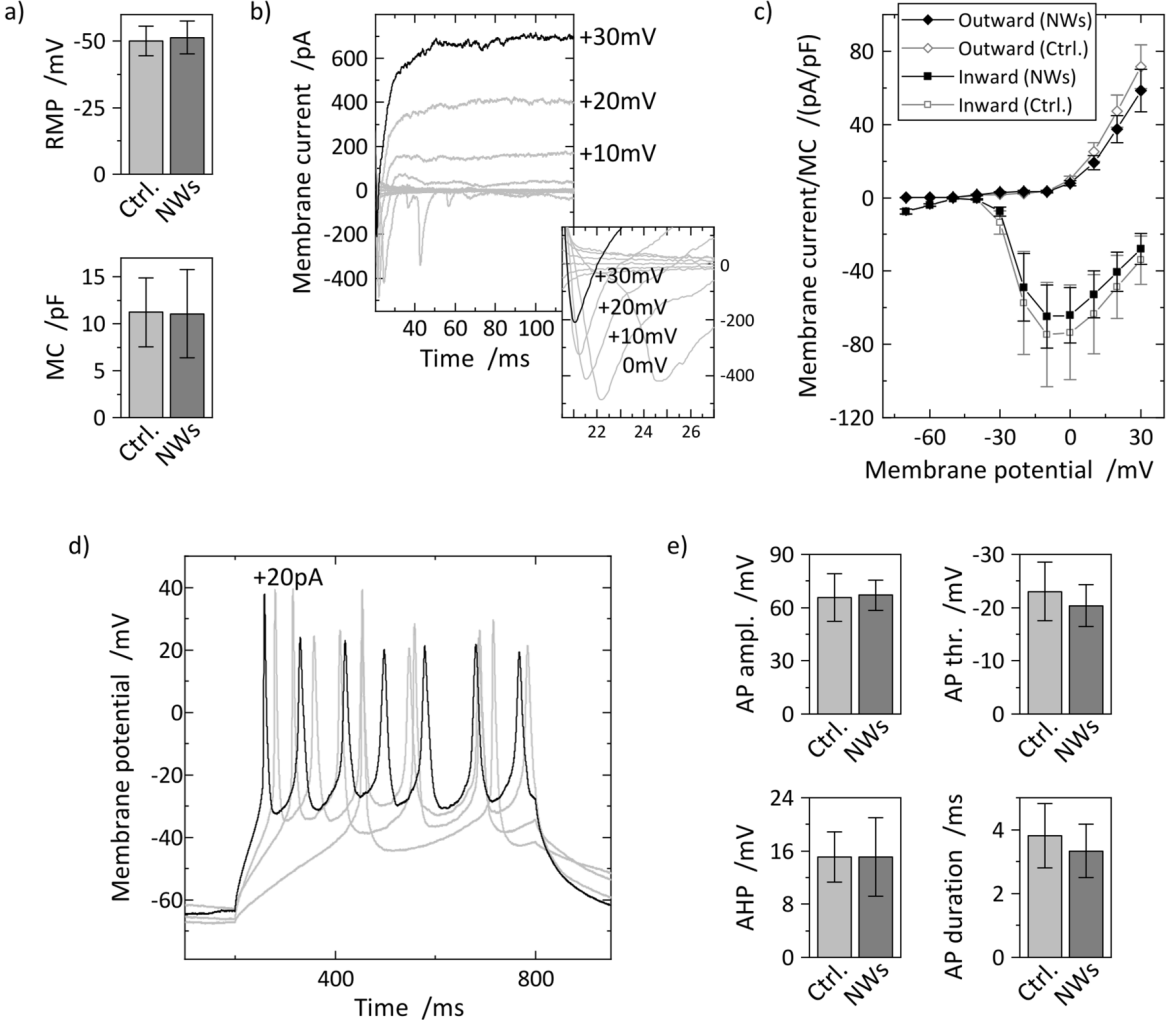
Electrophysiological characterization of human iPSC-derived neurons on NW substrates compared to control. (**a**) Quantification of passive membrane properties: resting membrane potential (RMP) and membrane capacitance (MC) of neurons on NWs and control. (**b**) Exemplary traces of early-inward (negative peaks) and late-outward currents (constant positive currents) of neurons cultured on NW substrates recorded in voltage-clamp mode with voltage steps of 10 mV from –70 to +30 mV. Inset: Zoom into early-inward currents. (**c**) Peak inward/outward currents normalized by MC plotted against membrane potential compared to control. (**d**) Typical action potential (AP) traces recorded from neurons on NWs. APs were stimulated by current injections from 5 to 20 pA in 5 pA steps. The firing frequency increases with increasing amplitude of the stimulus. (**e**) Analysis of the AP kinetics: AP amplitude from threshold to peak (AP ampl.), AP threshold (AP thr.), after hyperpolarization (AHP, overshoot after AP), and full width half maximum of the APs (AP duration). n(NWs) = 7, n(control) = 10, error bars are SDs, no statistical significances (Mann-Whitney U test: all P > 0.05, *cf.* Table S2 in the supporting material).

Finally, the electrophysiology and functionality of the neurons were tested using the patch clamping technique (Fig. 4a–e). The overall healthy state of the neurons cultured on NWs is validated by stable resting membrane potentials (RMPs) of –51.4 ± 6.1 mV shown in Fig. 4a. This value is similar to the control (no statistical significance, Mann-Whitney U test: P > 0.05, *cf*. Table S2) and normal or even above average compared to other human iPSC-derived neurons^45,52,56,57^. Moreover, the cell sizes including neuronal extensions on NWs and control are equal which is indicated by consistent membrane capacitances (MCs) of 11.2 ± 3.7 pF and 11.1 ± 4.7 pF on control and NW substrates, respectively (also Fig. 4a, no statistical significance, P > 0.05, *cf.* Table S2). In addition, the MCs are in accordance with literature^56,58,59^. The accurate interplay of sodium and potassium ion channels is approved by recordings of the membrane currents at different voltages (–70 to +30 mV, 10 mV steps) which show characteristic early-inward and late-outward currents (Fig. 4b). The peak currents normalized to the MC were plotted against the applied voltage (Fig. 4c) and are comparable to the control measurements and literature^4,52,59^. Neuronal identity is confirmed by recordings of action potentials (APs) evoked by current injection shown in Fig. 4d. AP amplitude (67.0 ± 8.6 mV), AP threshold (− 20.4 ± 4.0 mV), afterhyperpolarization (AHP, 15.1 ± 5.9 mV), and AP duration (3.3 ± 0.8 ms) match the control (Fig. 4e, no statistical significance, all P > 0.05, *cf.* Table S2) and are in agreement with other human iPSC-derived neurons in literature^56,57,59^.

In summary and conclusion, the human iPSC-derived neurons cultured on the densely-spaced spiky silicon NW substrates show excellent electrophysiological properties within only 20 days of differentiation despite the challenging substrate topology. The large-scale formation of neuronal networks on the NWs which is equal to control allows for novel approaches to study network properties under artificial constraints. Note, that the share of neurons is not only equal to control but even increased by 20% and thus would enhance the efficacy of future studies. The spiky shape of the nanowires renders the substrate suitable for the delivery of biomolecules or genes: such applications have already been shown using basic cell types like human umbilical vein endothelial cells (HUVECs) or human mesenchymal stem cells (hMSCs)^21,30,55^ and establishing more advanced cell types in the community would help to increase the clinical impact of the field. Moreover, the broad availability of silicon and many years of experience in both science and industry make the material a great candidate for large-scale and high-throughput purposes in neurodegenerative disease studies or pharmacological drug screenings. Thus, we believe that our findings are an excellent starting point for further studies using biological metamaterials featured by high aspect ratio nanostructured surfaces^60^.

## Methods

### Nanowire growth and sample preparation

The NW growth was previously described in Misra *et al.* and Zhang *et al.*^49,50^. In short: The NWs were grown on top of ZnO:Al coated silicon wafers. In the plasma-assisted VLS growth process, the growth of NWs is mediated by Sn nanoparticles, and the density of Sn nanoparticles can be controlled either by varying the initial thickness of the metal or by changing the parameters of H$_2$ plasma (to reduce the oxide layer and to form tiny catalyst droplets). Therefore, prior to loading into the PECVD chamber, Sn layers with nominal thicknesses from 1 nm to 5 nm were thermally evaporated on the substrates. Then, the Sn layers were exposed to H$_2$ plasma for 5 min at 180 °C, to transform them into separate Sn droplets. The chamber pressure, the RF power density and the flow rate were fixed at 600 mTorr, 50 mW/cm$^2$, and 20 sccm, respectively. Next, 6 sccm of silane (SiH$_4$) and 1.8 sccm of trimethylboron (1% TMB diluted in H$_2$) were introduced and the substrate temperature was raised to 400 °C to initiate the growth of NWs activated by the plasma, followed by a H$_2$ plasma etching to remove the remnant of Sn droplets. Typically, NWs grew around 1 µm long after 15 min deposition, showing a gradually tapered shape of around 80 nm diameters at their bases and less than 30 nm at their tips. NW and control samples (glass coverslips) were treated with an oxygen plasma, sterilized in 70% ethanol, and coated overnight at room temperature or for 1 h at 37 °C with Matrigel (Corning 354263, 1:150 in Knock Out DMEM (Life Technologies)).

### Cell culture

The human induced pluripotent stem cell (iPSC)-derived neurons were differentiated from human iPSC-derived small molecule neural progenitor cells (smNPCs)^52^. The neural progenitor cells were grown in basic N2/B27 medium (1:1 mixture of DMEM/F12 and Neurobasal medium, 1% penicillin/streptomycin/glutamine (100X), 1% B27 supplement without vitamin A (50x), 0.5% N2 supplement (100x), Life Technologies, Carlsbad, CA, USA) supplemented with 100 µM ascorbic acid (AA, Sigma-Aldrich, St. Louis, MO, USA), 0.5 µM SAG (Biomol, Hamburg, Germany), and 3 µM CHIR 99021 (Axon MedChem, Groningen, Netherlands). Differentiation was initiated by patterning medium (basic N2/B27 medium supplemented with 100 µM AA, 0.5 µM SAG, 1 ng/mL GDNF (PeproTech, Rocky Hill, NJ, USA), 1 ng/mL BDNF (PeproTech)) for 6 days and terminated in maturation medium (N2/B27 basic medium supplemented with 100 µM AA, 2 ng/mL GDNF, 2 ng/mL BDNF, 1 ng/mL transforming growth factor-$\beta$3 (TGF-$\beta$3, PeproTech), 100 µM dbcAMP (Sigma-Aldrich). The cells were kept at 37 °C with 5% CO$_2$ in a humidified atmosphere. 1×10$^6$ smNPCs were seeded to Matrigel-coated 6-well plate wells and kept in patterning medium for 6 days in vitro (DIV). Cells were split 1:5 using accutase (Sigma-Aldrich), seeded into 12-well plates containing the NW and control samples, and cultivated in maturation medium until the experiment. SEM imaging, confocal microscopy, and patch clamping were conducted 18–20 days after initiation of the differentiation. IF imaging was performed 1, 7, 11, and 15 days after plating on the NW and control samples. Data was collected from four independent differentiations from passages 22/24 and 24/26 from two thawings of vials containing the smNPCs. Two differentiations per experiment were used.

#### Ethics declarations

All experiments were conducted in accordance with the ethical statement in Reinhardt *et al.*^52^.

### Confocal microscopy

A Leica TCS SP8 in upright configuration (488 and 552 nm wavelength laser sources) was used for confocal microscopy. Cell membranes were stained in advance with a staining kit (neurite outgrowth staining kit, ex/em: 555/565 nm, Thermo Fisher). According to the manufacturer’s protocol, solutions were diluted in Dulbecco’s phosphate-buffered solution (DPBS), and cells were incubated in a 1× dye mixture (4% Formaldehyde added) for 15 min at 37 °C. The z-stacks were acquired in a 1× background suppression solution with a slicing distance of 172 nm. Processing and analysis of the z-stacks were conducted with the Leica software (Leica Application Suite X, v2.0.2.15022) or ImageJ/Fiji (v1.53c, https://downloads.imagej.net/fiji/archive/20191216-2110/fiji-win64.zip). For visualization, images were optimized in contrast, brightness, and false color, if applicable.

### Immunofluorescence staining

Samples were rinsed once with DPBS (Sigma-Aldrich), fixed in formaldehyde (4% in DPBS, Sigma-Aldrich) for 10 min, and again rinsed three times with DPBS. Covered with DPBS, samples were stored until imaging at 4 °C. Fixed cells were permeabilized and blocked for 45 min with 3% BSA (bovine serum albumin, Carl Roth, Karlsruhe, Germany), 0.1% Tween (Tween 20, Sigma-Aldrich), and 0.1% Triton-X (Triton X 100, Carl Roth) in DPBS prior to incubation with anti-MAP2 (microtubule-associated protein 2) primary antibodies (0.1% BSA, MAP2 1:500 in DPBS, mouse anti-MAP2 monoclonal antibody [Y113], Invitrogen, Cat. No. 13-1500) overnight at 4 °C. The cells were washed twice with DPBS and incubated with Alexa fluorophore-conjugated anti-mouse primary antibodies (0.1% BSA, Alexa 488 1:1000 in DPBS, goat anti-mouse IgG-Alexa Flour 488 polyclonal antibody, Invitrogen, Cat. No. A32723) for 1 h in the dark. Stained cells were kept in the dark and washed three times with Tween (0.05% in DPBS) for 5 min. The second washing step contained Hoechst 33342 as counterstain. If not further noted, all steps were conducted at room temperature. Per sample and point in time, 8–20 images were analyzed. The covered sample area per image size was 1282 µm × 853 µm. Images were analyzed using a custom analysis pipeline for CellProfiler 4.1.3 (https://cellprofiler-releases.s3.amazonaws.com/CellProfiler-Windows-4.1.3.exe). Hoechst images were used to identify nuclei. Vital cells were used to mask the MAP2 channel to identify marker positive cells. Images of identified vital cells were exported and clustering was analyzed using the SSIDC Cluster Indicator in the BioVoxxel toolbox in ImageJ/Fiji (v1.53c, https://downloads.imagej.net/fiji/archive/20191216-2110/fiji-win64.zip). Data was analyzed and graphs were generated with Origin (v.2021). In total, approx. 230.000 cells were analyzed.

### Viability assay

Viability was estimated in the CellProfiler pipeline by discrimination of bright nuclei and medium bright nuclei after Hoechst 33342 stain for the representation of dead and living cells, respectively. The procedure results in similar values with no statically significant differences (Mann-Whitney U test) compared to a test using Calcein AM and Propodium Iodide shown in the supporting information (Fig. S7).

### Scanning electron microscopy and focused ion beam milling

High-resolution images and cross sections were produced using scanning electron microscopy and focused ion beam milling with a Zeiss Crossbeam 550. Cells were prepared by fixation (4% Formaldehyde in DPBS, 10 min) and dehydration *via* a stepwise ethanol exchange. The samples were then critical point dried using Tousimis’ Autosamdri-815 and a 20 nm gold layer was sputter-coated to avoid charging effects.

### Electrophysiology

The patch-clamp setup consisted of an upright Nikon Eclipse FN1 microscope (objective: Nikon CFI TU Plan EPI ELWD 50× N.A. 0.60/W.D. 11.00 mm) and a HEKA EPC 10 USB patch-clamp amplifier with a red star headstage for trace recording. Patch-clamp pipettes (diameter: approx. 950 nm, resistance: 5–7 M$\Omega$) were manufactured from borosilicate glass capillary blanks (GB150T-8P, Science Products) using a Sutter P-2000 pipette puller. Polishing was conducted with a CPM-2 microforge (ALA Scientific Instruments). Samples were rinsed three times and covered with bath solution (140 mM NaCl, 2.4 mM KCl, 1.3 mM MgCl$_2$, 2.5 mM CaCl$_2$,10 mM 4-(2-hydroxyethyl)-1-piperazineethanesulfonic acid (HEPES), 10 mM D-glucose, pH 7.4) and pipettes were filled with pipette solution (125 mM potassium gluconate (K-gluconate), 10 mM NaCl, 1 mM Triethylene glycol diamine tetraacetic acid (EGTA), 4 mM magnesium ATP (MgATP), 10 mM HEPES, 10 mM D-glucose, pH 7.4). Patch clamping was conducted at room temperature. The Patch Master V2x80 software was used for data processing. A Bessel low-pass filter was set to 2.9 kHz and the capacitance and series-resistance were automatically compensated. Electrophysiological parameters were recorded and determined as follows. RMP: in current-clamp mode by keeping cells with zero current. MC: directly determined by the patch-clamp software. For APs, the first AP of an AP train at rheobase current was analyzed–AP height: threshold to peak. AHP: threshold to minimum after AP. AP threshold: membrane potential where the second derivative becomes different from zero (determined with Origin). AP duration: full-width half-maximum of the AP. Number of cells analyzed: n(NWs) = 7, n(control) = 10.

## Supplementary Information

Supplementary Information 1.
