# Supplementary material for: Robust neuronal differentiation of human iPSC-derived neural progenitor cells cultured on densely-spaced spiky silicon nanowire arrays
Source: Sci Rep. 2021 Sep 22;11:18819. doi: 10.1038/s41598-021-97820-4 (PMC8458299; doi:10.1038/s41598-021-97820-4)
Supplement: Supplementary file 1 — Supplementary Information 1. [file 41598_2021_97820_MOESM1_ESM.pdf]

Supporting information

Robust neuronal differentiation of human iPSC-derived neural progenitor cells cultured on densely-spaced spiky silicon nanowire arrays

Jann Harberts [1,*], Malte Siegmund [1], Matteo Schnelle [1], Ting Zhang [2], Yakui Lei [2], Linwei Yu [2], Robert Zierold [1,*] and Robert H. Blick [1,3]

[1] Center for Hybrid Nanostructures, Universität Hamburg, Luruper Chaussee 149, 22761 Hamburg, Germany
[2] School of Electronics Science and Engineering, Nanjing University, 210093 Nanjing, China
[3] Material Science and Engineering, College of Engineering, University of Wisconsin-Madison, Madison, WI 53706, USA
* Correspondence: jann.harberts@chyn.uni-hamburg.de, robert.zierold@chyn.uni-hamburg.de

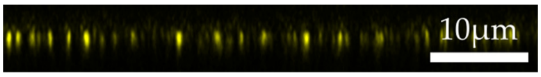

Figure S1: Cross-sectional CLSM image of silicon nanowires (yellow) without Matrigel coating.

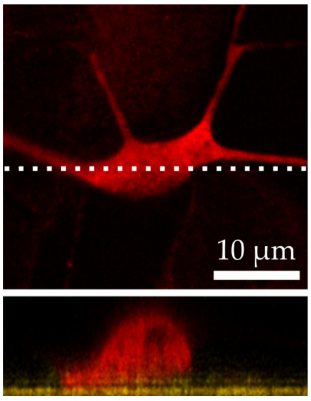

Figure S2: CLSM image including a cross section of a neuron cultured on a control substrate. The neuron appears in red, the reflection of the substrate is yellow.

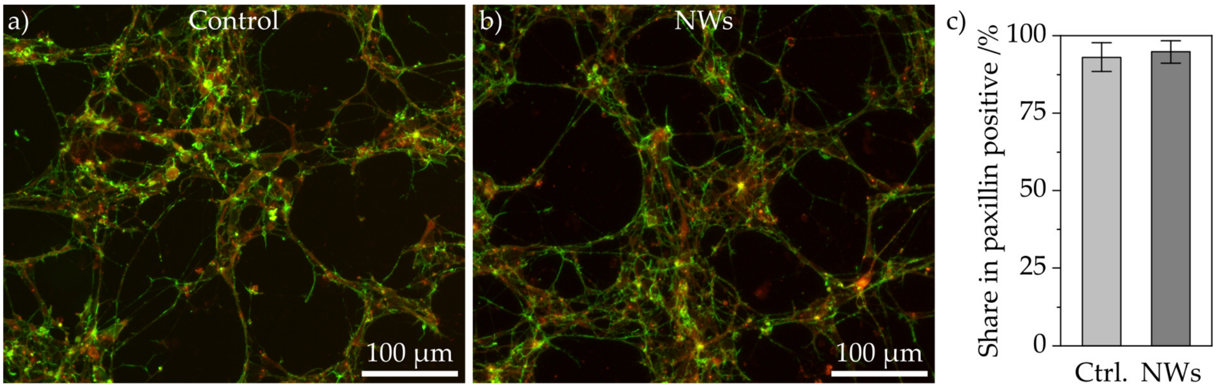

Figure S3: Comparison of f-actin/cytoskeleton (phalloidin, green) and paxillin/focal adhesions (red) of cells on NW and control substrates 11 days after plating. a) Cells on control substrate. b) Cells on NWs. c) Share in paxillin positive area in comparison with f-actin positive area. No statistical significance, Mann-Whitney U test, $P > 0.05$, error bars are SDs.

Samples were washed, fixated, blocked et cetera analogously to the IF staining procedure in the main manuscript. Besides, sells were stained with anti-paxillin primary antibodies (0.1% BSA, Pax 1:250 in DPBS, rabbit anti-Paxillin recombinant monoclonal Antibody [M13], Abcam, Cambridge, UK, Cat. No. ab32084) overnight at 4 °C. Samples were rinsed with DPBS two times. Secondary and Phalloidin staining was performed for 1 h in the dark with Alexa fluorophore conjugated anti-rabbit antibodies and phalloidin (0.1% BSA, Alexa 555 1:1000, PH: 2 drop /mL in DPBS, goat anti-rabbit IgG-Alexa Flour 555 polyclonal antibody, Thermo Fisher Scientific, Massachusetts, USA, Cat. No. A32732, phalloidin: ActinGreen Alexa Fluor 488 probe, Thermo Fisher Scientific, Cat. No. R37110).

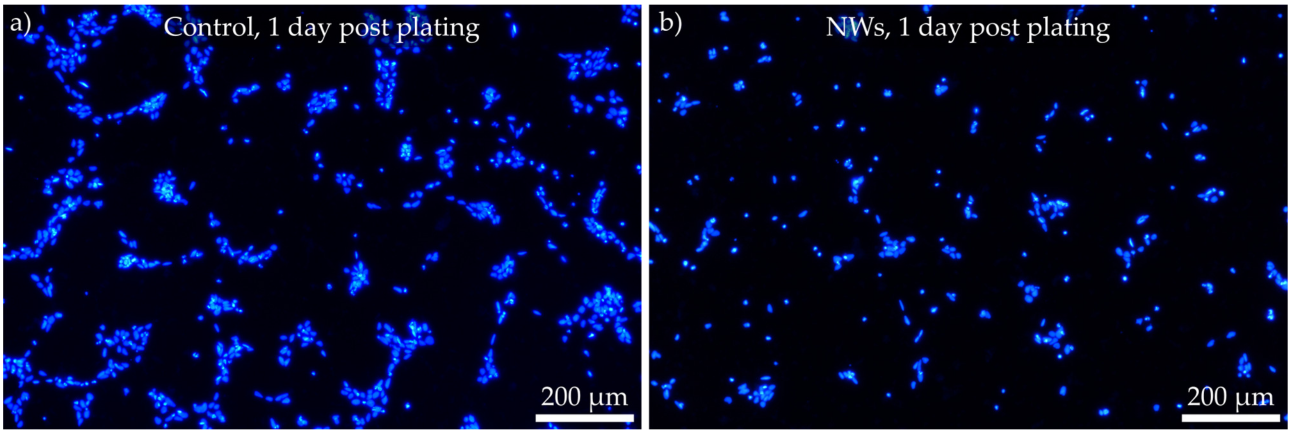

Figure S4: Exemplary images of Hoechst-stained cells (blue) on control (a) and nanowire arrays (b) one day after plating.

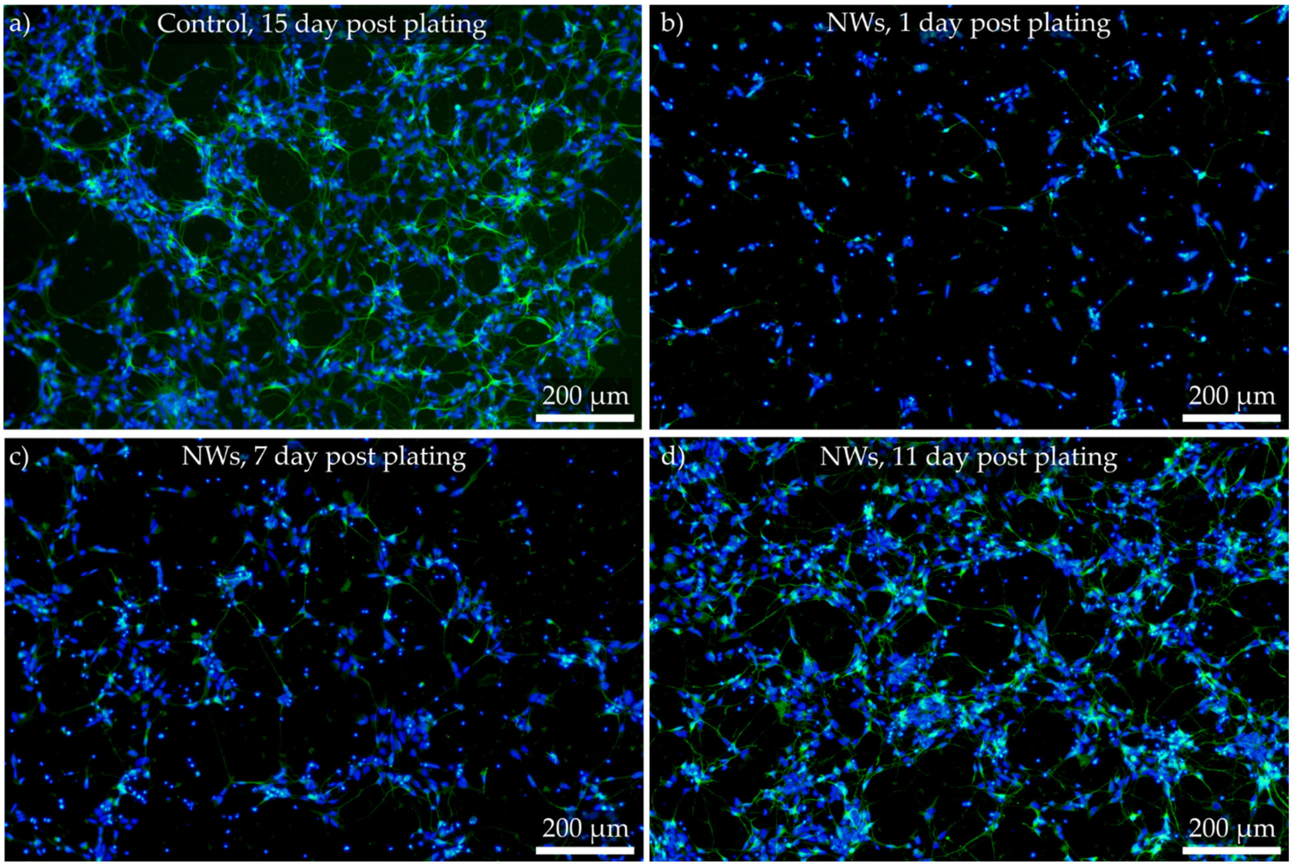

Figure S5: a) Exemplary image of neurons cultured on planar control substrate 15 days post plating labeled with anti-MAP2 (green) and Hoechst counterstain (blue). b-d) Neurons on NWs with anti-MAP2 labeling and Hoechst counterstain 1, 7, and 11 days post plating.

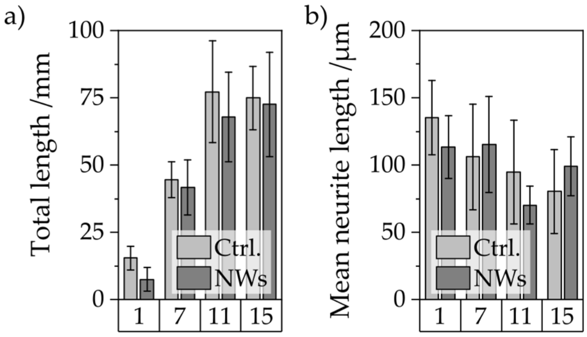

Figure S6: Total neurite length (a) and mean neurite length (b) of MAP2 positive cells determined by CellProfiler.

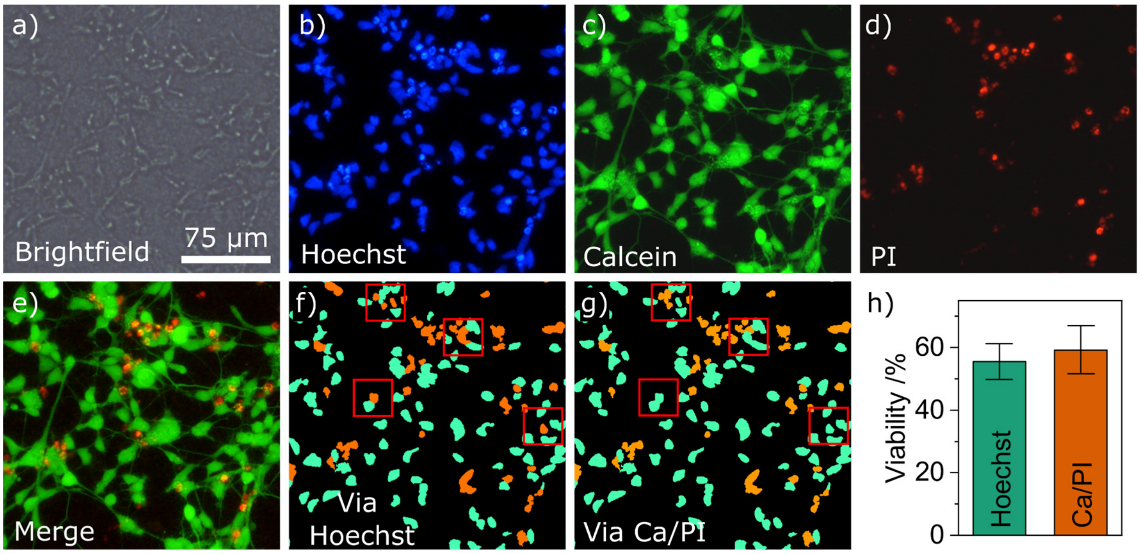

Figure S7: Comparison of estimating the viability by discrimination of vital and dead cells by brightness and by staining with Calcein and Propidium Iodide (PI). Exemplary widefield image (a) including the corresponding Hoechst (b), Calcein (c), and PI-stained (d) images including a merge (e). The analysis results from CellProfiler are compared to each other and the few differences are highlighted by red squares (f and g). The techniques deliver comparable results (55.5% vs 59.3%, bar chart in h) with no statistically significant differences (Mann-Whitney U test, $P = 0.16898 > 0.05$).

Table S1: Statistical significances of IF quantification in Figure 3. Mann-Whitney U test between NW and control substrates of cell number, single cells, cell viability, and MAP2 positive cells.

Parameter	Precise P	P threshold	Sig
Cell numer (1 DIV)	1.23893E-4	0.001	1
Cell numer (7 DIV)	0.33519	0.05	0
Cell numer (11 DIV)	0.07569	0.05	0
Cell numer (15 DIV)	0.00901	0.01	1
Viability (1DIV)	1.84916E-6	0.001	1
Viability (7DIV)	2.22169E-8	0.001	1
Viability (11DIV)	1.1515E-8	0.001	1
Viability (15DIV)	2.44257E-8	0.001	1
Single cells (1DIV)	1.29441E-5	0.001	1
Single cells (7DIV)	6.4152E-4	0.001	1
Single cells (11DIV)	1.51883E-5	0.001	1
Single cells (15DIV)	3.01902E-5	0.001	1
MAP2 positive (1DIV)	0.37442	0.05	0
MAP2 positive (7DIV)	0.80601	0.05	0
MAP2 positive (11DIV)	7.43885E-4	0.001	1
MAP2 positive (15DIV)	0.00103	0.01	1

Table S2: Statistical significances of electrophysiological properties in Figure 4. Mann-Whitney U test between NW and control samples of resting membrane potential, capacitance, AP amplitude, AP threshold, AHP, and AP duration.

Parameter	Precise P	P threshold	Sig
RMP	0.5362	0.05	0
MC	0.81253	0.05	0
AP amplitude	0.60088	0.05	0
AP threshold	0.19323	0.05	0
AHP	0.94138	0.05	0
AP duration	0.23879	0.05	0
